# Coping with the COVID‐19 pandemic by strengthening immunity as a nonpharmaceutical intervention: A major public health challenge

**DOI:** 10.1002/hsr2.1562

**Published:** 2023-09-13

**Authors:** Nizam Uddin, Thamina Acter, Md. Harun‐Ar Rashid, Akibul Islam Chowdhury, Effat Ara Jahan

**Affiliations:** ^1^ Department of Nutrition and Food Engineering, Faculty of Allied Health Science Daffodil International University Dhaka Bangladesh; ^2^ Department of Mathematical and Physical Sciences East West University Dhaka Bangladesh

**Keywords:** coronavirus‐2, COVID‐19, exercise, immune system, infection, nutrition, reduced stress

## Abstract

**Background and Aims:**

The global Coronavirus‐2 outbreak has emerged as a significant threat to majority of individuals around the world. The most effective solution for addressing this viral outbreak is through vaccination. Simultaneously, the virus's mutation capabilities pose a potential risk to the effectiveness of both vaccines and, in certain instances, newly developed drugs. Conversely, the human body's immune system exhibits a robust ability to combat viral outbreaks with substantial confidence, as evidenced by the ratio of fatalities to affected individuals worldwide. Hence, an alternative strategy to mitigate this pandemic could involve enhancing the immune system's resilience.

**Methods:**

The research objective of the review is to acquire a comprehensive understanding of the role of inflammation and immunity in COVID‐19. The pertinent literature concerning immune system functions, the impact of inflammation against viruses like SARS‐CoV‐2, and the connection between nutritional interventions, inflammation, and immunity was systematically explored.

**Results:**

Enhancing immune function involves mitigating the impact of key factors that negatively influence the immune response. Strengthening the immune system against emerging diseases can be achieved through nonpharmaceutical measures such as maintaining a balanced nutrition, engaging in regular exercise, ensuring adequate sleep, and managing stress.

**Conclusion:**

This review aims to convey the significance of and provide recommendations for immune‐strengthening strategies amidst the ongoing COVID‐19 pandemic.

## Key points

- Coronavirus‐2 outbreak is a global threat.
- Human immune system has the potential to fight against viral outbreaks effectively.
- Virus's mutation can reduce the efficacy of vaccines and drugs.
- Minimizing factors that adversely affect the immune response can improve immunity.
- The immune‐strengthening strategies are important during the COVID‐19 pandemic.

## 1 INTRODUCTION

**Figure 1:**
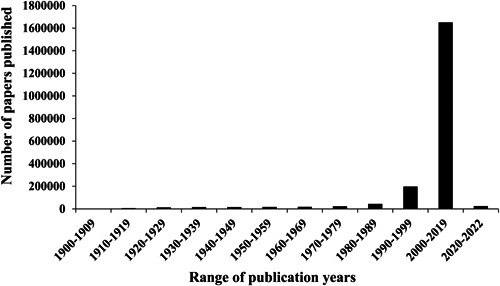
Number of papers published related to the search keywords “immunity and infection” in Google Scholar site.

It has been nearly 3 years since the onset of the coronavirus disease‐2019 (COVID‐19) pandemic worldwide. This pandemic originated as an infectious respiratory illness triggered by the severe acute respiratory syndrome coronavirus‐2 (SARS‐CoV‐2) virus in Wuhan, China, since December 2019.^1^ On March 11, 2020, the World Health Organization (WHO) classified this ailment as a pandemic, owing to its rapid transmission across over 229 countries and territories. As of June 18, 2023, there have been a staggering 768 million documented infections and 6.9 million confirmed fatalities.^1^, ^2^, ^3^ At present, multiple COVID‐19 vaccines, such as Pfizer‐BioNTech, Moderna, Johnson & Johnson, AstraZeneca, and Sinovac, have been formulated to trigger the body's immune system into generating antibodies against the virus.^4^, ^5^ Nevertheless, no vaccine can offer complete protection against a virus. The efficacy of a vaccine is also influenced by the response of the immune system, as well as the type of vaccine and the virus itself. Even after receiving vaccination, it remains crucial to uphold non‐pharmaceutical interventions (NPIs) such as wearing masks, implementing lockdowns, temporarily closing educational institutions, practicing social distancing, adhering to home quarantine, maintaining proper hand hygiene, bolstering immunity, enhancing indoor humidity, and ventilating indoor spaces. These measures have been adopted and continue to be practiced in numerous countries worldwide.^6^, ^7^, ^8^, ^9^, ^10^, ^11^ Among these various NPIs, sustaining a healthy immune system is deemed the most potent defense for navigating through such viral outbreaks.^12^, ^13^, ^14^ While there is presently no evidence supporting protection against a second infection for individuals with COVID‐19 and existing antibodies,^15^ the intricate interplay between the virus and the immune response warrants consideration. Due to a pronounced immunological connection between immunity and infection, whose significance has been discussed in the literature (see Figure 1), the potential role of the SARS‐CoV‐2 virus in interacting with the host's immune system may be pivotal in influencing the susceptibility, progression, and eventual outcome of COVID‐19 disease.^16^, ^17^, ^18^ Tian et al.^19^ demonstrated that individuals with compromised immune systems exhibit heightened vulnerability to SARS‐CoV‐2 infection and face an elevated risk of fatality. For instance, older individuals and those afflicted by noncommunicable diseases, such as diabetes, heart disease, hypertension, lung ailments, and obesity, are at an elevated risk of contracting COVID‐19. Consequently, they may be susceptible to the development of severe complications associated with the disease due to their compromised immune systems.^20^ Given the context of the COVID‐19 pandemic, characterized by a deficiency in effective preventive and curative treatments, Chowdhury et al.^21^ proposed that enhancing the immune response could be regarded as a supplementary approach for combating the coronavirus during the ongoing crisis. Hence, enhancing the immune response during the initial asymptomatic phase of SARS‐CoV‐2 infection has been deemed essential, necessitating the individual to be in a state of good health. The objective of this review is to furnish readers with essential health insights by imparting comprehensive understanding about bolstering immune defenses, potentially offering protective or ameliorative effects against COVID‐19 infection.

## 2 METHODS

As depicted in Figure 1, a strong immunological correlation between immunity and infection is evident. The review's research question aims to acquire a comprehensive understanding of the role of inflammation in COVID‐19 and the alleviation of inflammatory burden through the enhancement of immune defense. A thorough search of pertinent literature regarding immune system functions, the role of inflammation against viruses like SARS‐CoV‐2, and the connection between nutritional interventions and inflammation and immunity was conducted using databases such as PubMed, Google Scholar, and Science Direct. The search period for “immune system functions” extended from 1960 to 2020, while the exploration of “nutritional interventions” in relation to inflammation and immunity encompassed the years 2000–2020. The investigation into “inflammation's role against viruses such as SARS‐CoV‐2” specifically focused on the year 2020. The search results underwent screening to identify studies relevant to the research question. Essential information was then extracted from the chosen studies to synthesize and present the findings in a coherent narrative.

## 3 RESULTS AND DISCUSSION

### 3.1 Major functions of immune cells

The immune system of a living organism (host) serves as a defense mechanism comprised of different specialized cells and organs. These components work together to combat infections or diseases caused by a variety of agents, referred to as pathogens, ultimately protecting the host.^22^, ^23^ The most crucial immune cells include immunoglobulins, anti‐inflammatory cytokines, lymphocytes, neutrophils, natural killer (NK) cells, monocytes, T cells, and B cells.^23^

NK cells and CD8+ T lymphocytes display high cytotoxicity and the ability to migrate within tissues. White blood cells, known as leukocytes, are pivotal to the immune system. Among them, phagocytes (e.g., neutrophils) consume foreign invaders known as antigens, while lymphocytes help to recognize and eliminate these threats. This recognition and destruction process involves B lymphocytes (B cells) and T lymphocytes (T cells). B cells generate antibodies to tag antigens, while T cells aid B cells and phagocytes in neutralizing antigen‐antibody complexes. The presence of specific antibodies is critical for establishing immunity against corresponding diseases.

### 3.2 Stages of immune response

1. Innate immunity:
  The innate immune system comprises special types of cells like NK cells, macrophages, dendritic cells, neutrophils, cytokines, and interleukins. This system acts as the primary physical and chemical barrier,^17^ akin to the skin, defending the human body from foreign invaders.^24^
2. Adaptive/active immunity:
  The long‐lasting immunity, known as active immunity, develops gradually over a person's lifetime.^24^, ^25^ The adaptive immune system includes T lymphocytes, B lymphocytes, and their products—antibodies and cytokines.^17^ This immunity can arise from disease exposure or vaccination, triggering the body's immune response for immediate antibody production to combat the disease. Macrophages and lymphocytes mediate cellular adaptive immunity, while antibodies mediate humoral adaptive immunity.^26^, ^27^
3. Passive immunity:
  The temporary immunity, termed passive immunity, occurs when a person receives antibodies from external sources like antibody‐rich blood products (e.g., immunoglobulin) or from a mother to her newborn baby.^24^, ^25^

### 3.3 Mechanism of developing immunity against virus

**Figure 2:**
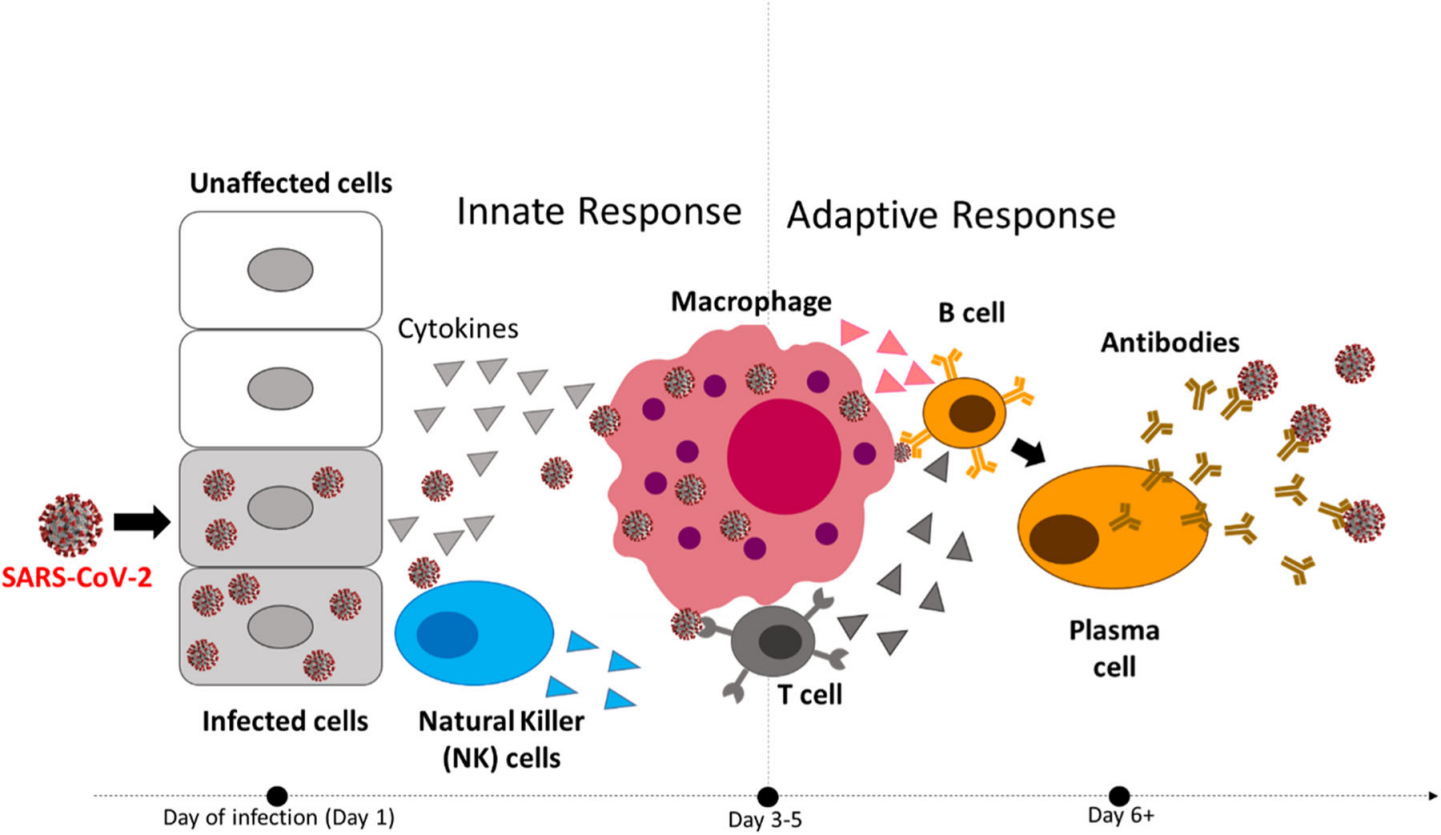
Mechanistic illustration of developing immunity against virus.

A robust immune defense system involves a swift reaction to foreign viruses and the development of immunological chronic memory system.^23^ The process of developing immunity against foreign substances like viruses is illustrated in Figure 2 and summarized below:

1. The SARS‐CoV‐2 virus, a foreign invader, triggers inflammation in infected cells as it replicates, generating numerous virus particles within the cell. Over a short span, these inflamed cells become distressed. In response, specific cells of the host's immune system release cytokines, which are small protein‐like signaling molecules.
2. At first, these cytokines activate various NK cells referred to as “first‐line responders.” These NK cells are responsible for eliminating infected cells and capturing newly produced virus particles within the cell.
3. Subsequently, second‐line responders are mobilized, leading to the formation of macrophages that swallow virus particles. These macrophages then break down into B and T cells, ultimately producing viral fragments as antigens on their surfaces.
4. In such situations, B and T cells protect infected cells by attaching antibodies to viral fragments or antigens. These antibodies form clusters around the antigens.
5. Frequent involvement of T cells is essential to control the rate of virus particle replication and enhance antibody production within the host cell.

### 3.4 Immunity status to the novel SARS‐CoV‐2 virus

**Figure 3:**
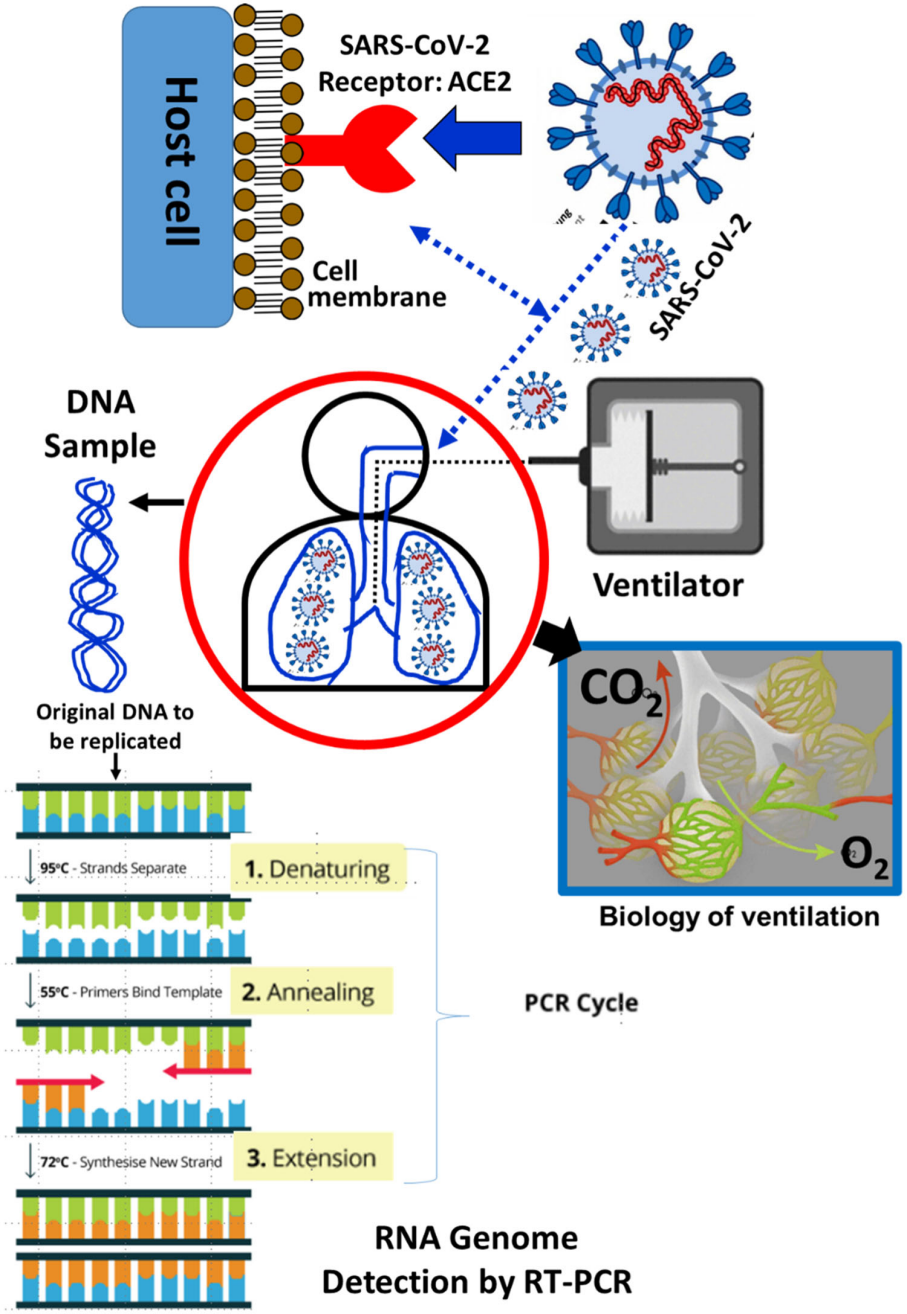
The key features of SARS‐CoV‐2 infection showing its clinical characteristics, diagnosis, and supportive treatment.

People worldwide have encountered limited immunity to the novel SARS‐CoV‐2 virus, despite its genetic resemblance to prior pandemic coronaviruses like the severe acute respiratory syndrome‐related coronavirus (SARS‐CoV) and the Middle Eastern respiratory syndrome coronavirus (MERS‐CoV).^28^ The virus's envelope spike protein (S) primarily infects host cells by binding firmly to angiotensin‐converting enzyme‐2 (ACE2) receptors in the lower airways.^28^, ^29^ Subsequently, it targets other cell types, including cardiac, intestinal, renal, and vascular cells, through viral replication and dissemination.^29^ The key features of COVID‐19 caused by the SARS‐CoV‐2 virus are depicted in Figure 3.

Unfortunately, the activation mechanism of the host immune response to the SARS‐CoV‐2 infection still remains completely unknown due to the virus's novelty. Information about the extent of immunological memory formed among B and T cells after exposure to the infection, as well as the duration of this memory, is yet to be clarified.^23^ Nonetheless, studies have examined the impact of host immunity on the severity of SARS‐CoV‐2 infection. For instance, the role of innate immunity in COVID‐19 pathogenesis and acute respiratory distress syndrome (ARDS), a critical condition characterized by severe lung inflammation and fluid accumulation in air sacs, has gained prominence. This is attributed to the recruitment of inflammatory cells like neutrophils and macrophages, along with elevated levels of reactive oxygen species (ROSs).^17^

### 3.5 Role of inflammation in COVID‐19

Inflammation plays a significant role in COVID‐19's pathogenesis and progression. Typically commencing with flu‐like symptoms,^30^ and the disease may manifest asymptomatically or range from mild to severe.^31^ The infection carries a substantial inflammation burden.^32^ Nonetheless, in certain instances, the inflammatory response may escalate and become excessive after several days of coronavirus exposure, resulting in a phenomenon termed cytokine storm or hyperinflammation.^18^ Cytokines, which are small signaling molecules governing immune responses, become overproduced during this storm. Proinflammatory cytokines like interleukin‐6 (IL‐6) and tumor necrosis factor‐⍺ (TNF‐⍺) are among them. Notably, C‐reactive protein, interleukin, and various immune mediators play roles in the disease's progression.^33^ This uncontrolled cytokine release can lead to widespread inflammation and potential damage to organs such as the lungs, heart, liver, and kidneys. The severe inflammation observed in critical COVID‐19 cases contributes to the development of ARDS, which can cause respiratory failure, necessitating mechanical ventilation. Moreover, it can lead to endothelial dysfunction, damaging the cells lining blood vessels and giving rise to complications like strokes, heart attacks, and pulmonary embolism.

Therefore, a strong immune system is thought to mitigate cellular inflammation, viral replication, and the spread of SARS‐CoV‐2.^34^, ^35^ The effectiveness of immune responses in COVID‐19 patients might be compromised by factors like cytokine storms and diminished antibody production in older individuals. The impact of comorbidities such as obesity, diabetes, and high blood pressure on the immune defense system during SARS‐CoV‐2 infection remains controversial. Enhancing immune defense could potentially offer protective or ameliorative benefits against COVID‐19 infection.

### 3.6 Strategies of improving immunity against COVID‐19

**Figure 4:**
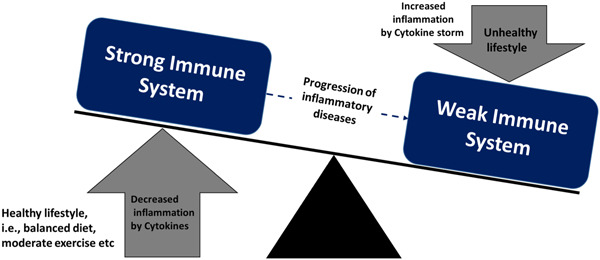
Interaction between immunity‐contributing risk factors and immune response to infection.

Several factors can influence an individual's immune competence and susceptibility to infection. Lifestyle, age, health status (nutritional deficiency, physical exertion), stress, sex, medications, sleep deprivation, smoking, and exposure to environmental pollutants play a role. Hesarya and Akbari^36^ proposed a hypothesis highlighting groups vulnerable to COVID‐19, such as the elderly, smokers, males, and those with chronic medical conditions. Zabetakis et al.^37^ illustrated the interaction between immunity‐contributing risk factors and the immune system (Figure 4). Inflammatory molecules can disrupt immune responses due to existing risk factors, increasing infection susceptibility. Conversely, reducing risk factors can enhance the immune response against infection. Therefore, it's crucial to improve immune function by mitigating major contributing factors altering the immune response.

Nutrition closely links to inflammation^38^ and immunity.^39^ Jayawardena et al.^40^ first systematically reviewed nutritional interventions, like vitamins, minerals, nutraceuticals, and probiotics for preventing viral respiratory infections such as COVID‐19. Later, several research groups underscore the importance of balanced nutrition during the COVID‐19 crisis.^41^, ^42^, ^43^, ^44^, ^45^, ^46^, ^47^, ^48^, ^49^ An effective immune system relies on proper diet, reducing inflammation and oxidative stress,^50^ which modulate the immune response.^51^, ^52^ COVID‐19 triggers an immediate immune response with cytokine release and primer adaptive T and B cell responses. This response can combat infection, though severe cases may arise occur due to immune dysfunction.^53^ Patients with severe infection exhibit elevated plasma levels of IL‐2, IL‐7, IL‐10, macrophage inflammatory protein 1α (MIP1α), IP‐10 and tumor necrosis factor (TNF).^54^ Nutrition aids in reducing inflammation. Additionally, Silveira et al.^17^ suggest regular exercise to strengthen immune functions during the pandemic.

**Scheme 1:**
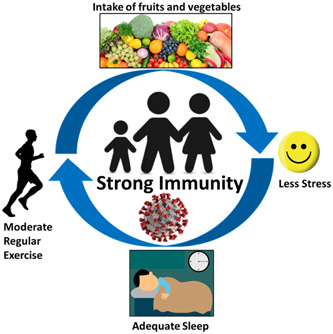
Strategy to boost up the overall immunity to combat COVID‐19 infection.

However, the concept of “immune boosting” is often misleading and used for marketing unproven products and therapies.^55^, ^56^ Scheme 1 outlines a combined approach involving nutritional interventions, exercise, and stress relief as a potential strategy to boost up the overall immunity and combat COVID‐19 infection in normal individuals.

#### 3.6.1 Optimal nutrition and balanced diet

##### Nutrition and the immune response

It is well known that human nutrition involves incorporating essential nutrients into food to maintain good health and nutrients are necessary for survival, growth, and reproduction of a living organism. Macronutrients, including carbohydrates, fats, proteins, fiber, and water, are vital for energy generation, tissue repair and growth, requiring larger amounts (grams or ounces).^57^ Micronutrients, including minerals (iron, zinc, and selenium) and vitamins (A, B6, B12, C, D, E and folate) are required in smaller amounts (milligrams or micrograms) playing critical roles in the biochemical and physiological functions of cellular processes.^57^, ^58^ Both macronutrients and micronutrients are crucial for the immune system's proper functioning.

**Figure 5:**
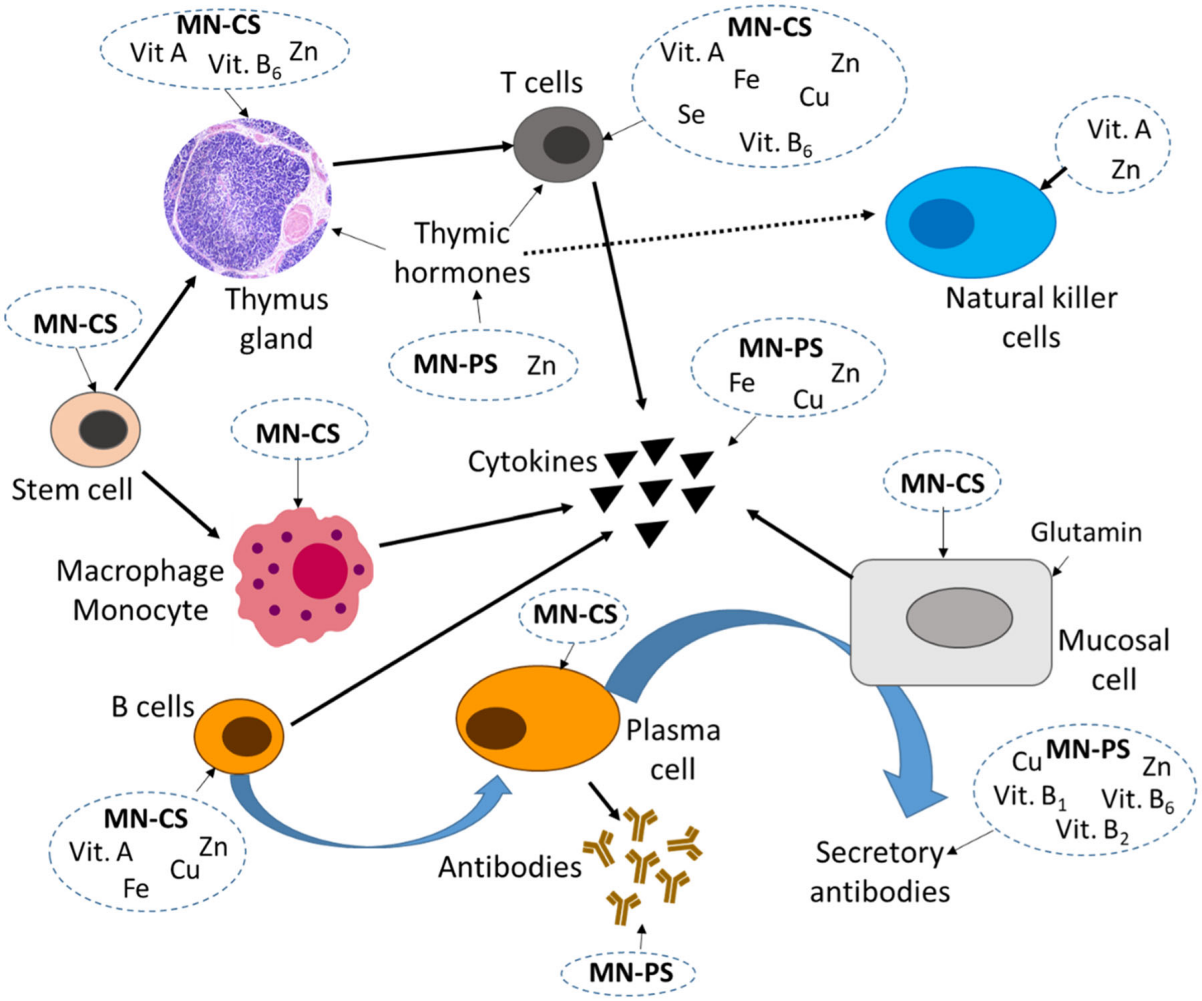
Involvement of nutrients in immune system function, where MN‐CS and MN‐PS denote multiple nutrients for cellular synthesis and multiple nutrients for protein synthesis respectively.

Table 1 displays major nutrients and their roles in the immune system. To enhance diets and develop survival‐oriented nutritional interventions during any pandemic, fundamental knowledge of food and nutrition can be applied at various levels—individual, family, community, institutional, and national.^92^ In 1968, the WHO highlighted the interaction between infection and malnutrition.^93^ Several studies also reveal connections between nutrition and immune responses.^22^, ^94^, ^95^ The immune system significantly benefits from balanced consumption of essential nutrients, influencing gene expression, cell activation, and modification (Figure 5). Nutrient intake alterations may stem from food preferences, varying quantities, or nutrient supplement use. Inadequate essential nutrient intake results in nutritional or dietary deficiency, while excessive intake leads to “over nutrition” or nutritional toxicity.^96^

**Table 1:** Some of the major nutrients with roles implicated in immune function.

Nutrients	Major immunity functions	Recommended dietary allowance (RDA)	Good food source
Protein: (Composition: Amino Acids, that is, arginine, glutamine, taurine, creatine, carnosine, anserine and 4‐hydroxyproline)	Proinflammatory effects by animal‐based proteins, anti‐inflammatory effects by plant‐based proteins,^59^, ^60^, ^61^ optimal production of antibodies,^62^ lowering postprandial lipogenesis and inflammation,^63^ reducing postmeal glycemic response,^64^ increasing intestinal immunoglobulin levels,^65^ and promoting gene transcription during cell proliferation^66^	0.8 g/kg body weight^67^	Beans and peas, legumes, eggs, fish, poultry, red meat, whey protein^67^
Lipids: (Composition: saturated and unsaturated fatty acids, omega‐3 or α‐linolenic acid, eicosapentaenoic acid and docosahexaenoic acid, omega‐6 or arachidonic acid)	Higher and lower high‐sensitivity C‐reactive protein (hs‐CRP) levels are linked to the consumption of saturated FAs and polyunsaturated FAs respectively^68^; anti‐inflammatory capability of omega‐3,^69^ proinflammatory and increased TNF‐α, IL‐6, and hs‐CRP levels of unhealthy trans‐fatty acids.^70^ Increased onset of allergic, autoimmune, and metabolic conditions due to the imbalance of saturated/unsaturated fatty acids, and omega‐6/omega‐3 fatty acids.^71^	Omega‐6 and omega‐3, with a ratio of 1:1–4:1^67^	Omega‐3: fish and seafood such as salmon, mackerel, and tuna.^67^
Carbohydrates: (Composition: Monosaccharides, that is, glucose; disaccharides, that is, sucrose; oligosaccharides, that is, raffinose; polysaccharides, that is, cellulose; sugar alcohols, that is, sorbitol, mannitol.	High consumption may cause an overload of mitochondrial capacity, free radicals, glycemic index‐induced acute hyperglycemia,^72^ and levels of TNF‐α and IL‐6.^73^	225–325 g/day^74^	Less processed, low‐glycemic load foods, such as vegetables, fruit, nuts, seeds, and whole grains.^67^
Fiber: (Composition: Undigested carbohydrates)	Potential mediation of the intestinal microfloral immunity due to lactic acid bacteria or bacterial products in the intestine^75^	38 g/day (adult men), 25 g/day (adult women)^67^	Soluble fiber: barley, oatmeal, beans, nuts, and fruits such as apples, berries, citrus fruits, and pears; Insoluble fiber: whole grains, wheat cereals, vegetables such as carrots, celery, and tomatoes.^67^
Water: (Composition: minerals such as calcium, fluoride, iron, potassium, and sodium	Transporting all nutrients to each organ system through blood stream; increasing lymphatic draining by clearing out any foreign invaders and other waste materials; contributing to muscle tension, headaches, low serotonin production, and digestive issues.^76^	A minimum of half of body weight in ounces of water^76^	Purified water, herbal teas, low‐sugar electrolyte powder, coconut water.^76^
*Vitamin A and carotenoids*	Leading to production of isotretinoin for inhibiting the regulation of ACE2 receptor; inability for viral attachment and infection; decreasing the susceptibility in contracting COVID‐19.^77^, ^78^	700 µg/day (men) and 600 µg/day (women)^79^	Carrots, sweet potatoes, spinach, broccoli, bell peppers, and mangoes.^79^
*Vitamin E*	As antioxidant and strengthening the body's natural defense against infection.^79^	4 mg/day (men), 3 mg/day (women)^79^	Eggs, tuna, salmon, nuts, seeds, and avocado.^79^
*Vitamin C*	Stimulating the production of antibodies^79^ and strengthening the immune system against harmful pathogens.^80^	3 ~ 10 g/day^79^	Liver, oyster, citrus fruits, guava, strawberries, pineapple, broccoli, and tomato.^79^
*B‐Vitamins: thiamine (vit.B1), riboflavin (vit.B2), niacin (vit.B3), pantothenic acid (vit.B6), biotin (vit.B7), folic acid (*vit.B9), cobalamin *(vit.B12)*	Playing a crucial role in the healthy balance of the immune system^81^	B1: 1 mg/day (men), 0.8 mg/day (women)^79^	Peas, fruits such as bananas and oranges, nuts, whole grain breads, milk, and eggs.^79^
*Vitamin D* (D2 or D3)	Powerful immune regulator expressed by the majority of immune cells (B and T lymphocytes, macrophages, and monocytes,^82^ ability to alter the susceptibility to infection,^83^, ^84^ and supporting healthy bone.^85^	400 IU/day; 12,000 IU/month^79^	Fatty fish, enriched dairy products.^79^
*Vitamin K*	Promoting proper blood clotting, strengthening bones, and helping in protecting cells from oxidative damage.^79^	1 μg/day for each kg of their body weight^79^	Green leafy vegetables such as broccoli and spinach, vegetable oils, and cereal grains.^79^
*Iron*	Fundamental element for normal development of the immune system, required for several biochemical reactions of immune and nonimmune cells, and pathogens.^86^	8.7 mg/day (men >18 years), 14.8 mg/day (women aged 19–50 years), 8.7 mg/day (women >50 years)^79^	Liver, red meat, red kidney beans, chickpeas, nuts, dried apricots, fortified breakfast cereals, and soybean flour.^79^
*Zinc*	Inhibiting viral replication by preventing viral membrane fusion and improving antiviral response and symptomatology.^87^, ^88^	More than 75 mg/day^79^	Oysters, beef, chicken, baked beans, pumpkin seed, almonds, peas.^79^
*Copper*	Copper deficiency may reduce interleukin‐2 and T cell proliferation.^79^	Adults aged 19–64 years need 1.2 mg/day	Oyster, shellfish, seeds, nuts, whole bran cereals, whole grain products
*Selenium*	Initiating immunity and regulating excessive immune responses and chronic inflammation^89^	75 μg/day for men, 60 μg/day for women	Turkey, eggs, chicken, milk, tofu, sunflower seed, whole grain cereals
*Phosphorus*	Supporting immune functions and providing a barrier against potential pathogens by maintaining a stable microbial ecosystem in the gastrointestinal tract.^90^	550 mg/day^79^	Red meat, dairy foods, fish, poultry, bread, brown rice, oats^79^
*Potassium*	Regulating the heartbeat and ensuring proper function of the muscles and nerves, and vital for synthesizing protein and metabolizing carbohydrates.^79^, ^91^	Adults (19–64 years) 3500 mg/day^79^	Bananas, broccoli, brussels, sprouts, beans, pulses, nuts and seeds, fish, beef, chicken, and turkey^79^

Both deficiency and toxicity of specific nutrients impair the immune response, affecting humoral and cell‐mediated immune functions, causing catabolic response, shifting infection risk, protein‐energy malnutrition (PEM), altering cytokine production, reducing antibody affinity, suppressing secretory immunoglobulin A (IgA) antibody response, diminishing lymphocyte activation, and phagocyte dysfunction.^97^ Hence, the immune response serves as a functional indicator of an individual's health status.

##### Importance of balanced diet to combat COVID‐19

**Figure 6:**
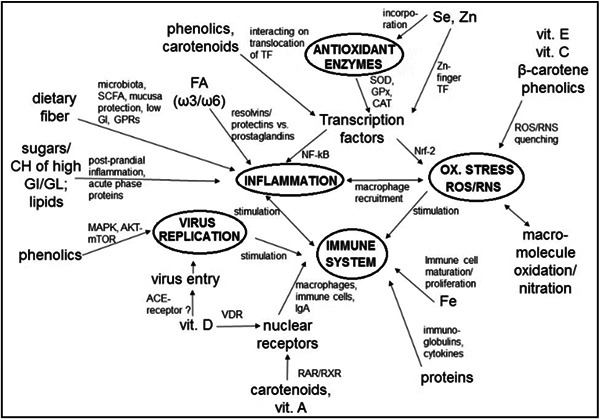
A detailed schematic diagram showing interactions among dietary constituents, immunity, and viral infection.

A balanced and consistent long‐term dietary pattern, featuring nutritious and healthy foods, can boost the immune system by managing oxidative stress and inflammatory processes. This, in turn, may decrease infection risk and promote health during the COVID‐19 period.^98^, ^99^ An association between diet and the trans‐membrane ACE2 level exists.^100^ ACE2 facilitates the SARS‐CoV‐2 virus's entry into host cells (Figure 6). Figure 6 demonstrates that a diet rich in protein, micronutrients like vitamins A, C, D, and E, B vitamins, zinc, selenium, iron, and phytochemicals can have a positive impact on the immune system. Notably, a high‐saturated fat diet elevates blood ACE levels,^101^ while a diet abundant in vegetables can inhibit ACE levels.^102^ Consequently, the immune response may weaken due to reduced macrophage activation during antigen production.^103^ Thus, a compromised immune system with diminished virus‐fighting ability could heighten the host's susceptibility to coronavirus infection.

The COVID‐19 outbreak has led to global food insecurity. Factors contributing to this issue during lockdowns include panic buying and storing foods, reduced purchasing power due to income loss, higher prices of certain foods, limited access to markets, restricted imports, and increased wastage of fresh foods due to transportation constraints.^104^ Lockdowns and social confinement have induced changes in individuals' food habits, potentially disrupting balanced diets. These shifts toward imbalanced and unhealthy diets, marked by nutritional deficiencies and empty calories, have manifested as “food cravings.” Reduced consumption of fresh fruits, vegetables, and high‐quality protein‐rich foods like meat/fish, along with increased intake of saturated fats, sugary foods, carbohydrates, and snacks, are notable examples.^105^

Consequently, various chronic noncommunicable diseases, including obesity, diabetes, cardiovascular disease, and chronic respiratory disease, which exacerbate COVID‐19 prognosis (i.e., mortality), have emerged. These conditions are considered comorbidities, amplifying the risk of severe COVID‐19 complications.^106^ Vulnerable groups at higher risk of viral infection include immunosuppressed individuals, particularly the elderly and those with pre‐existing comorbidities.^107^ Geographical variation, distinct complications, and mortality during the COVID‐19 pandemic may result from diets with nutritional deficiencies.

Hence, nutrition takes precedence in the current COVID‐19 era. Bolstering individual immune systems through proper nutrition and balanced diets is crucial for resilience and maintaining robust immune function, addressing nutrient deficiencies, managing diet‐related chronic diseases, and triggering immune responses against pathogens like COVID‐19. Ensuring recommended calorie intake and immune‐supportive nutrients becomes particularly important during times of heightened infection risk, such as the current pandemic, benefiting all age groups, especially the vulnerable populations.

##### Suggested food intake during COVID‐19

Foods known for their immune‐boosting qualities, abundant in nutrients like magnesium, copper, zinc, iron, folate, and vitamins A, B6, B12, C, and D, can aid in enhancing immune resilience. Healthy foods and their roles in strengthening the immune system during COVID‐19 are outlined in Table 2. Table 2 reveals that plant‐based foods are reservoirs of numerous bioactive micronutrients and antioxidants. Fruits and vegetables are well‐known for their antioxidant, vitamin, mineral, and phytochemical content, contributing to their anti‐inflammatory and other beneficial properties that can aid in immunity regulation and aging prevention.^114^ However, excessive consumption of any food could lead to nutrient imbalances by displacing other essential foods. Wallace et al. recently examined dietary recommendations for enhancing individual health.^122^ To balance nutritional needs and enhance overall well‐being during the COVID‐19 outbreak, the WHO has proposed specific dietary guidelines. These include consuming four servings of fruits/day, five servings of vegetables/day, a mix of whole grain cereals (180 g), and a variety of meats and beans (160 g).^123^ The WHO/Europe and WHO/Eastern Mediterranean Regional Office (EMRO) have provided general guidance to support healthy eating during self‐quarantine and isolation^123^:

- Assessing food intake to reduce the panic buying behavior and avoid food waste.
- Planning mealtimes.
- Engaging in family meals to foster healthy eating and strengthen family relationships.
- Including fresh and unprocessed foods, such as 2 cups of fruits/day, 2.5 cups of vegetables/day, 180 g of whole grains/day, and foods from animal sources like meat, fish, eggs, and milk.
- Ensuring sufficient fiber intake in all meals for a well‐functioning digestive system.
- Consuming an adequate amount of water (8–10 cups/day) to facilitate nutrient transport and waste elimination.
- Moderating fat and oil consumption, opting for white meat (e.g., poultry) and fish, and incorporating unsaturated fats (e.g., nuts, olive oil, soya oil, canola oil, sunflower oil, corn oils).
- Adhering to safe food handling practices to maintain a healthy diet and prevent food contamination.

**Table 2:** Suggested healthy foods for improving the immune system to combat COVID‐19.

Name of healthy foods	Category	Major composition	Major role to improve immunity with reference
Turmeric	Spice	Curcumin	Antioxidant and anti‐inflammatory properties^108^
Broccoli	Vegetable	Vitamins A, C, and E, sulforaphane, and phytochemicals	Antioxidant properties^109^
Spinach	Vegetable	Vitamin A (β‐carotene), Vitamin C, flavonoids, carotenoids	Increasing the disease‐fighting cells in the body and helping in proper immune function^110^
Strawberries	Fruit	Vitamin C and bioactive phytochemicals	Reducing cell damage caused by free radicals and thus strengthening the immune system^111^
Sunflower seeds	Plants	Phosphorus, magnesium, copper, calcium, iron, selenium and vitamin E	Reducing cell damage caused by free radicals and thus boosting the immune system^112^
Garlic	Herb	Sulfur‐containing compounds	Antiviral and antibacterial properties; reducing the risk of heart disease and blood pressure; boosting the immune system by fighting against common cold and infections^113^
Almond	Dried food	Vitamin E, antioxidants, manganese, fiber	Protecting against oxidative stress; reducing cholesterol levels; helping in strengthening the natural killer cells and lymphocytes^110^
Lemon/Orange	Fruit	Vitamin C	Helping the immune cells to function properly^114^
Kiwi	Fruit	Vitamins C, A, B6, B12, and E, potassium, calcium, iron, folate	Providing the body a nutritional boost^114^
Mushrooms	Vegetable	Vitamin D	Reducing the production of proinflammatory compounds and thus lowering the risk for respiratory tract infections^115^
Milk and milk products	Dairy	Essential vitamins and minerals such as calcium, zinc, selenium, magnesium, and potassium	Maintaining a healthy immune response during fighting against viruses^116^
Yogurt	Probiotics/Fermented foods	Nonpathogenic and nontoxic bacteria, namely Lactobacillus and Bifidobacterium,	Promoting and restoring a healthy gut microbiome and treating digestive problems, playing a crucial role in immunomodulation and maintaining a strong immune defense against infections^117^
Papaya	Vegetable and fruit	Vitamins B and C, potassium, folate, digestive enzyme known as papain	Anti‐inflammatory property, boosting immune system^110^
Tomato	Vegetable	Potassium; carotenoids; ascorbic acid; lutein; α‐, β‐, and γ‐tocopherols, and conjugated flavonoids	Culinary role in the diet, lowering risk of stomach and rectal cancers^110^
Bell pepper	Vegetable	Vitamins C and K, carotenoids, and flavonoids	Preventing prostate cancer, cancer of the bladder, cervix, and pancreas; reducing inflammation found in arthritis and asthma^110^
Cucumber	Vegetable	Vitamin C, pro vitamin A as β‐carotene, fiber, potassium, silicon	Antioxidant activity and antiproliferative activity in relation to human liver cancer cells, connecting tissue, such as skin, hair, and nails by silicon^110^
Onion	Vegetable	Flavonoids, flavonols like isorhamnetin, kaempferol myricetin, and quercetin;	Strong antiviral activity^113^
Carrot	Vegetable	β‐carotene	Inhibiting RNA replication and thus decreasing hepatosteatosis induced by hepatitis C virus (HCV), reducing inflammation by increasing leukocytes in the body^110^
Beetroot	Root vegetable	Phytochemicals and bioactive compounds, flavonoids, polyphenols; inorganic nitrate; minerals	Reducing oxidative stress and inflammation^118^
Radish	Vegetable	Vitamin C, fiber, potassium and folate	Treating Helicobacter pylori infection and blocking gastric tumor formation^119^
Lettuce	Vegetable	Vitamin C, folate, fiber and pro vitamin A (β‐carotene)	Antioxidant properties, protecting the macula lutea of the eye and the skin against the same photo‐oxidative damage, other potential health‐promoting bioactivities include antiallergic, anti‐inflammatory, antimicrobial, and anticancer properties^120^
Celery	Vegetable	Potassium and sodium, some vitamin C, a small amount of vitamin A as β‐carotene, sodium, potassium, calcium, and fiber, bioactive compounds	Cardiovascular benefits and anti‐inflammatory properties^110^
Oats, wheat bread, barley malt	Whole grains and refined grains	Carbohydrate, β‐glucans, arabinoxylan, tocols, resistant starch, and polyphenols	Exhibiting antiviral effects by reducing inflammation and alleviating symptoms, reducing stress hormones during chronic disease or SARS‐CoV‐2 infection and limiting immune depression^121^

For a healthy nutritional routine during quarantine, Muscogiuri et al.^45^ suggested adopting the Mediterranean Diet, which includes olive oil, fresh fruits and vegetables, protein‐rich legumes, fish, and whole grains with moderate red meat consumption.

##### Foods to avoid during the COVID‐19 pandemic

Minimizing negative health effects during the COVID‐19 quarantine involves avoiding certain unhealthy foods and practices. These include:

###### Excess salt and sugar

Foods like sodas, soy sauce, fish sauce, and sweets contain high levels of salt and sugar. Overconsumption can increase cytokine secretion and worsen inflammation in blood vessels. WHO recommends limiting salt and sugar intake to ≤5 g (approximately 1 teaspoon/day) during.^124^

###### Excess fat

Saturated fats found in fatty meat, butter, coconut oil, cream, cheese, ghee, and lard can cause inflammation in fatty tissues. Daily caloric intake from fats should not exceed 10%.^124^ Industrially produced trans fats, found in processed foods like fast foods, should also be avoided.

###### Excessive alcohol

Excessive alcohol consumption can disrupt the immune system by absorbing immunogenic material in the intestine and impacting granulocytopoiesis.^125^ Alcohol‐induced malnutrition impairs nutrient absorption, utilization, storage, and excretion, indirectly affecting nutritional immunomodulation. Overconsumption may lead to inadequate intake of essential nutrients like proteins, vitamins (folate, thiamine, A, B6), and minerals (zinc). Alcohol can also affect humoral and cellular immunity due to abnormalities in immune cells like macrophages, lymphocytes, mononuclear phagocytes, and cytokines (TNF, IL‐1, IL‐6).^126^ Therefore, limiting alcohol intake is necessary.

###### Smoking

Smoking can have adverse impacts on the respiratory system due to its superimposition on other stressors. It has been reported that smoking is associated with the increased severity of COVID‐19.^127^ The WHO recommends tobacco cessation during the pandemic.

#### 3.6.2 Physical exercise

##### Immune responsive mechanism of exercise in humans

It has been reported that regular physical exercise offers immune defense enhancements and metabolic health improvements in younger individuals through cumulative acute immune system changes.^128^ This stimulates cellular immunity, lowering systemic inflammatory process risks.^128^ For the elderly, habitual exercise reduces oxidative stress, increases PHA‐induced lymphocyte proliferation,^129^ and delays immunosenescence onset.^130^ Immunosenescence involves age‐related functional decline in innate and adaptive immunity, potentially reducing host response to antibodies and increasing susceptibility to infections and diseases in old age.

**Figure 7:**
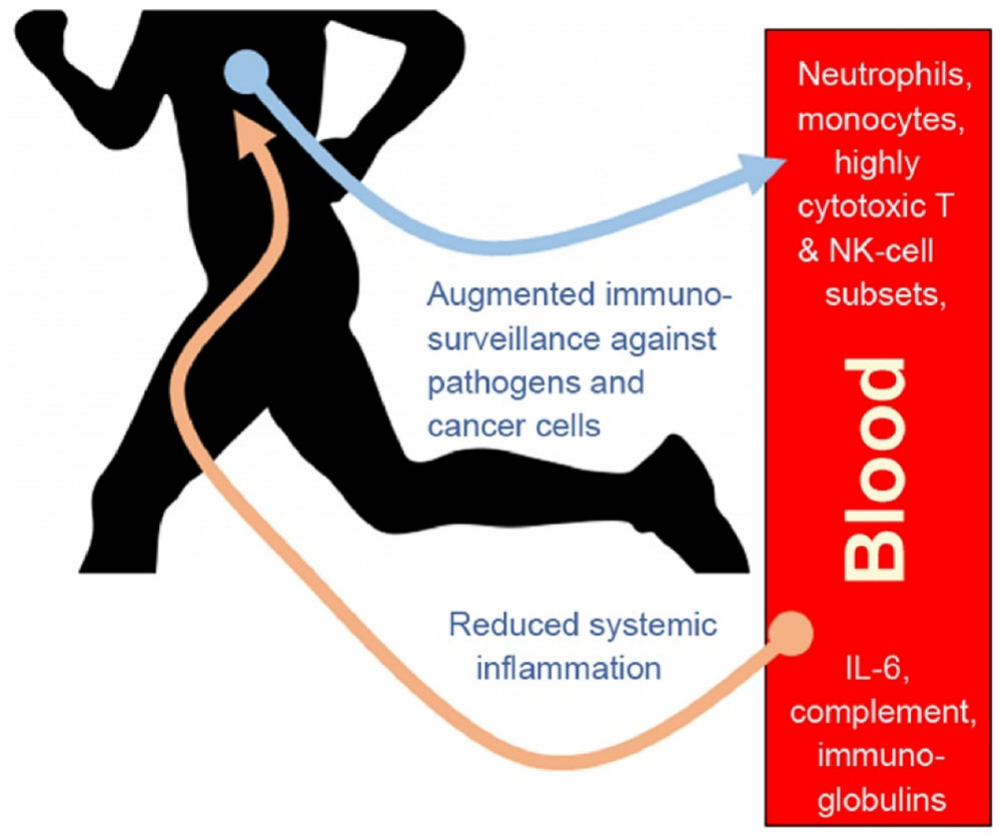
Benefits of performing acute exercise.

Figure 7 depicts the advantage of acute exercise, which can lead to preferential mobilization and redistribution of highly cytotoxic CD8+ T lymphocytes and NK cell subsets in the blood compartment.^131^, ^132^ This effect can decrease inflammation even in the presence of other diseases.^133^, ^134^, ^135^ Moreover, these temporary exercise sessions may elevate the production of the anti‐inflammatory cytokine IL‐6, enhancing glucose and lipid metabolism.^136^ Other benefits of regular exercise include improved vaccine response, enhanced cardiorespiratory function, leukocyte telomere length improvements, reduced exhausted T cells, enhanced neutrophil phagocytic activity, and positive effects on glucose, lipid, and insulin metabolism.^17^, ^137^ Therefore, regular physical exercise, particularly of light‐to‐moderate intensity, can be a nonpharmaceutical measure to positively impact immunomodulation. It can be an essential component alongside dietary control for preventing psychological, physical, and metabolic disorders. Exercise affects the immune system and is influenced by infection's impact on exercise performance. These factors hinge on exercise intensity, duration, and immune response.^138^

##### Role of physical exercise in fighting COVID‐19

**Figure 8:**
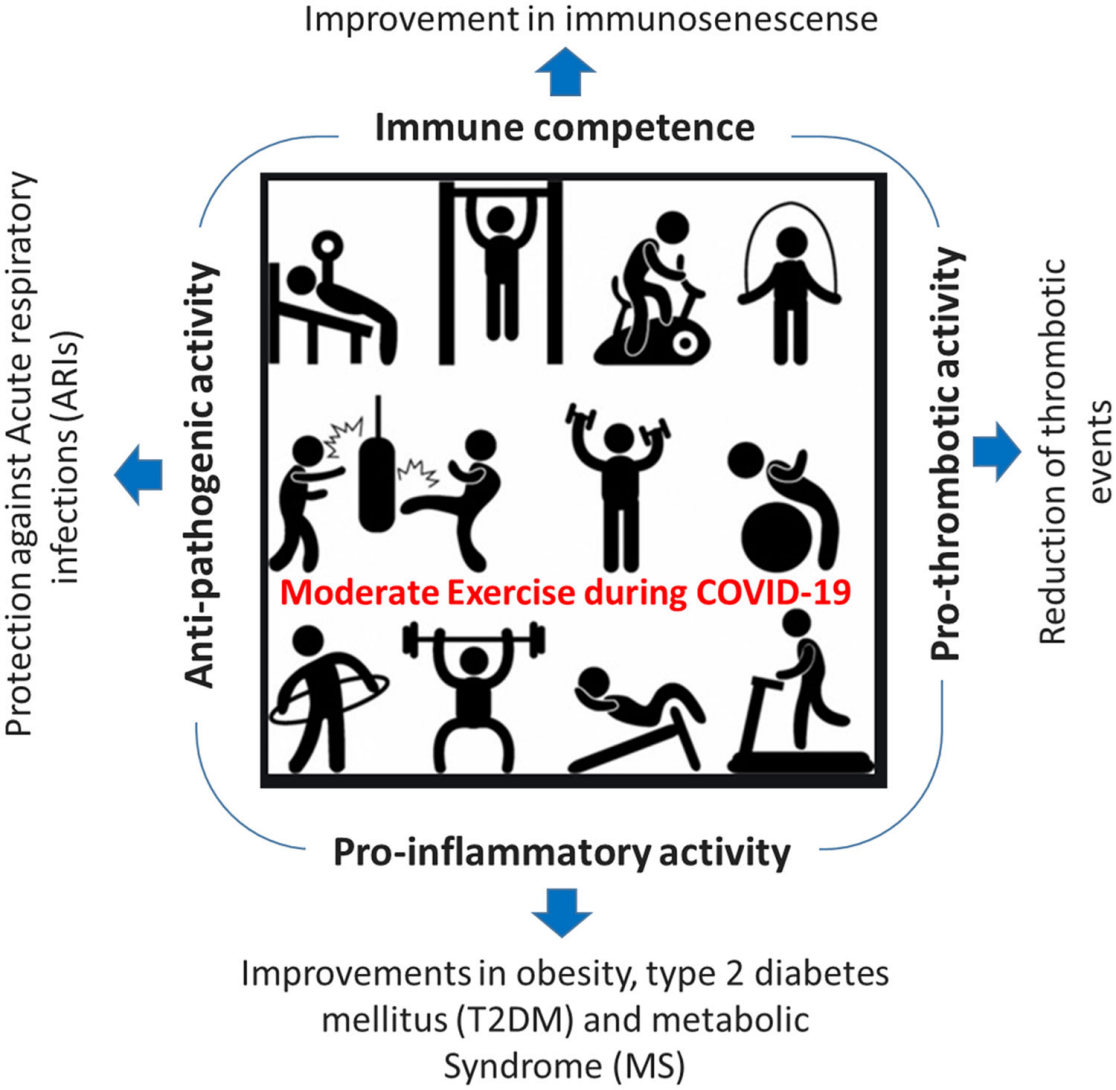
Role of moderate physical exercise during COVID‐19 fighting against SARS‐CoV‐2 virus.

During the COVID‐19 pandemic, various countries have implemented quarantine measures like confinement and social isolation to limit the spread of the latest SARS‐CoV‐2 virus. As a result, individuals have become more susceptible to adopting a sedentary lifestyle, leading to behavioral and physiological shifts, including eating disorders and weight gain.^139^ Figure 8 illustrates the role of physical exercise in activating immune functions to combat the emerging coronavirus. The connection between SARS‐CoV‐2 infection's immunopathogenesis and the individual's physical and health status underscores the significance of maintaining regular exercise, even amidst quarantine.^17^, ^26^, ^140^

As previously explained, engaging in regular and moderate physical activity for less than 60 min is advantageous in lowering the host's susceptibility to upper respiratory tract infections. This is achieved through improved immunosurveillance, increased exchange of white blood cells between the circulatory system and tissues, heightened antipathogenic activity of tissue macrophages, and enhanced recirculation of immunoglobulins, inflammatory cytokines, and other vital immune cells.^128^, ^141^, ^142^ A positive correlation has been reported between regular moderate exercise and improved outcomes during flu‐like viral infections like the 1998 Hong Kong flu.^143^ Furthermore, previous studies have established a direct association between regular exercise and reduced mortality from pneumonia and influenza.^17^, ^143^

In general, the most vulnerable populations to SARS‐CoV‐2 infection are the elderly^17^ and individuals who are overweight or obese,^144^ often with additional health conditions. For instance, Damiot et al.^145^ observed similar levels of TCD4 + and TCD8 + lymphocytes in both elderly and younger individuals who maintained regular physical activity during the COVID‐19 pandemic. For obese individuals, maintaining metabolic control of glucose, lipid levels, and blood pressure through regular exercise is vital to impede the new coronavirus's cell entry, enhance infection response, and prevent cardiovascular complications.^146^ Physical exercise may also address coagulation disorders during the COVID‐19 outbreak by reducing tissue damage and enhancing the production of immunomodulators like anti‐inflammatory agents, antioxidants, and endothelial activation inhibitors.^147^, ^148^ Regardless of whether a population falls within a COVID‐19 risk group, promoting regular physical exercise as a preventive measure during the pandemic is essential to fortify immunity against the SARS‐CoV‐2 virus and maintain robust health and strong immune system.

##### Suggested physical exercises during the COVID‐19 pandemic

In the context of COVID‐19, practicing outdoor physical activities in sports clubs and gyms appears to be a challenge due to social isolation, quarantine, and restricted environments.^149^ However, numerous indoor alternatives can be adopted to maintain physical exercise routines.

For instance, moderate indoor activities encompassing aerobic and strength exercises for 20–30 min, three to four times a week, have been recommended by the WHO and the American College of Sports Medicine (ACSM).^17^ This recommendation corresponds to at least 150 min per week for adults and 300 min per week for children and adolescents. The home environment is an ideal space for indoor physical exercise during the pandemic. Adults can incorporate exercise into their daily routines through activities such as cleaning, cooking, organization, and utilizing exercise equipment like treadmills, stationary bicycles, and rowing machines.^150^ Among aerobic options, activities like walking indoors, utilizing stairs, and jumping rope can be effective.^17^ For strength training (callisthenics), exercises like squats, sit‐ups, push‐ups, and yoga can be practiced.^150^ The WHO/Europe has developed a set of home‐based exercises to facilitate physical activity and systematic exercise at home.^151^ Furthermore, technological resources like online programs and applications provided by physical education professionals are also available for safe and convenient use.^150^ To adhere to infection‐preventive measures, outdoor activities like walking, running, cycling, and gardening can be practiced.^152^ Nevertheless, it's important to avoid engaging in physical exercises before or during an infectious condition, such as influenza or COVID‐19, without consulting a healthcare professional. It's also crucial to recognize the importance of exercising at appropriate levels of intensity.

#### 3.6.3 Relief of mental stress

##### Immune responsive mechanism of stress in human

It has been reported that stress response can be regulated through a range of behavioral and physiological adaptations to mitigate various pathologies.^153^, ^154^ Behavioral adaptations may include awareness, euphoria from engaging in social activities like aerobic exercise, laughter, music, and dancing, which contribute to a happier life. Additionally, improved cognition involving cognitive processes such as thinking, knowing, remembering, judging, and problem‐solving, as well as enhanced analgesia for pain relief, can occur. Physiological adaptations may involve factors like respiratory rate, metabolism, and immunity.^155^

**Figure 9:**
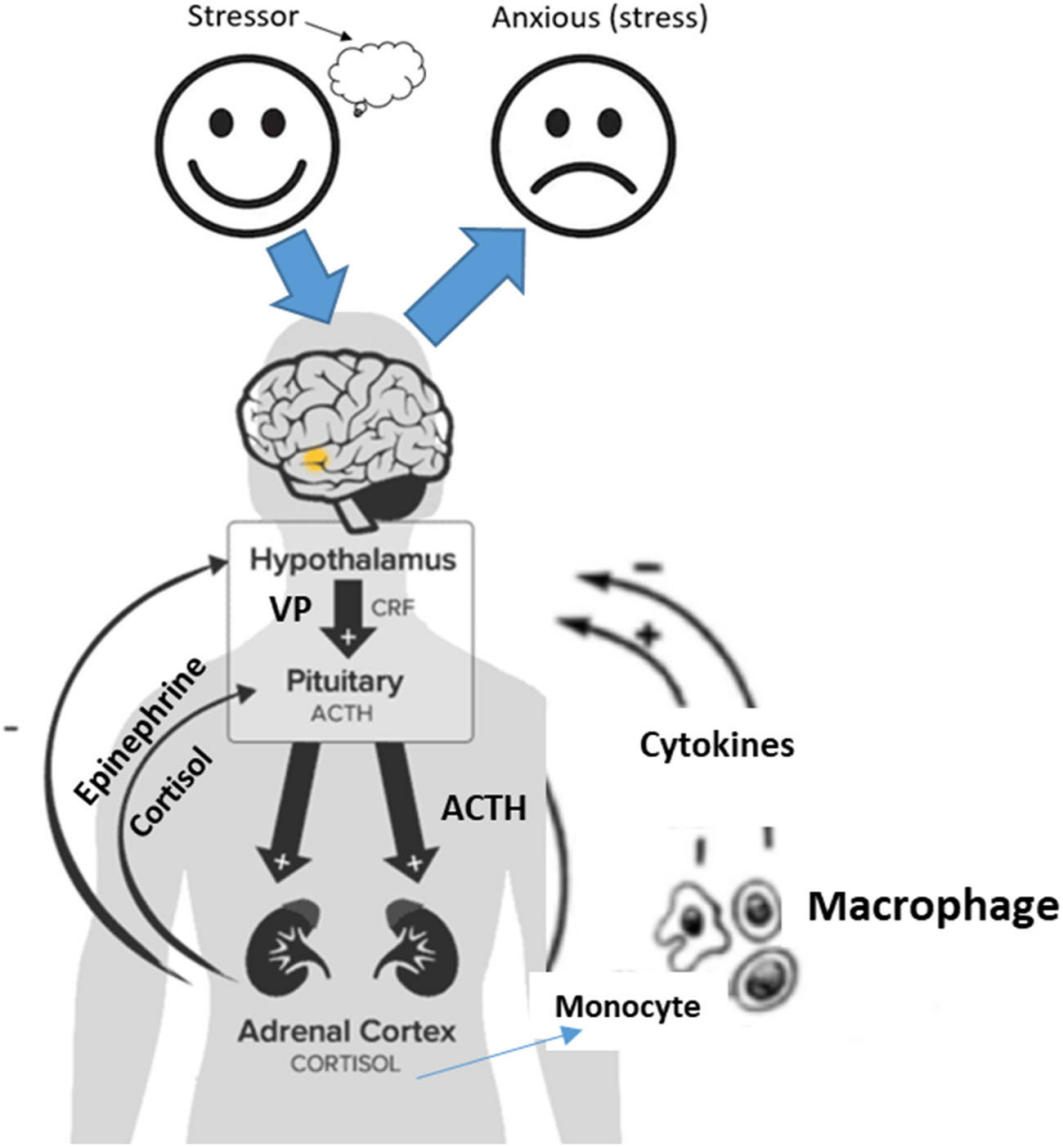
Immune responsive mechanism of stress response in humans.

Figure 9 illustrates the principal stress response effectors found in the hypothalamus of the central nervous system, pituitary gland, and the adrenal gland, collectively known as the hypothalamic‐pituitary‐adrenal (HPA) system.^156^, ^157^ The HPA system plays significant roles in regulating adaptive responses to stress.^156^, ^158^, ^159^ and thus can be affected significantly by inflammatory disease such as infections, resulting in the release of cytokines that directly stimulate the hypothalamic release of corticotropin‐releasing factor (CRF) or corticotropin‐releasing hormone (CRH) and vasopressin (VP). CRH enhances the release of alarm chemicals like epinephrine and norepinephrine from the adrenal medulla, which collectively promote the secretion of adrenocorticotropic hormone (ACTH) from the anterior pituitary gland. ACTH, in turn, prompts the release of glucocorticoids, such as cortisol, from the adrenal gland for several hours following exposure to stressors, potentially inhibiting various components of the immune response. A hypothesis by Munck et al.^160^ suggests that the pituitary‐adrenal stress response may suppress excessive inflammatory processes by frequently mobilizing cytokines and related inflammatory mediators, as supported by the study by Sternberg et al.^161^ Thus, maintaining a healthy HPA system is vital for supporting balanced cortisol levels. Notably, it has been observed that young men and older women produce higher cortisol levels in response to stress.^162^

##### Suggested ways for reducing stress during the COVID‐19 pandemic

Quarantine or social confinement, along with the news of COVID‐19‐related issues, can lead to stress in the community population. This can result in psychological challenges during times of “social distancing” and “self‐isolation.” Athletes, for example, may experience depression due to sudden changes in their training routines.^163^, ^164^ To alleviate stress during this pandemic, the following suggestions can be considered:

1. Engaging in regular moderate physical exercise during the pandemic can positively impact psychological well‐being by reducing perceived stress and anxiety disorders.^17^, ^165^
2. Techniques like meditation, deep breaths, stretching, and relaxation can counteract sedentary habits during the COVID‐19 pandemic.^17^ The WHO/Europe has proposed relaxation methods.^151^
3. Trained healthcare professionals can offer routine counseling and psychosocial support to individuals at high risk for COVID‐19 infection and those with chronic illnesses, aiding in their mental well‐being.
4. Ensuring adequate sleep (~7 h/day) is crucial to prevent stress resulting from sleep disturbances during the pandemic.^166^ One hundred and twenty consuming sleep‐promoting amino acids and foods containing serotonin and melatonin, such as almonds, bananas, cherries, oats milk, and dairy products, at dinner can support sound sleep.^167^

## 4 CONCLUSION

In addition to available vaccines or proven treatments during the COVID‐19 pandemic, enhancing immune defense becomes a top priority as a nonpharmaceutical preventive measure and treatment approach. Strategies like balanced diet, regular moderate exercise, and stress relief are significant for strengthening the immune system during pandemics. This promotes a healthy immune response to effectively counteract future viral and bacterial pathogens.

## AUTHOR CONTRIBUTIONS

**Nizam Uddin**: Conceptualization; data curation; investigation; methodology; project administration. **Thamina Acter**: Methodology; resources; supervision; visualization; writing—original draft. **Md. Harun‐Ar Rashid**: Resources; supervision; writing—original draft; writing—review and editing. **Akibul Islam Chowdhury**: Investigation; writing—review and editing. **Effat Ara Jahan**: Investigation; writing—review and editing.

## CONFLICT OF INTEREST STATEMENT

The authors declare no conflict of interest.

## TRANSPARENCY STATEMENT

The lead author Nizam Uddin affirms that this manuscript is an honest, accurate, and transparent account of the study being reported; that no important aspects of the study have been omitted; and that any discrepancies from the study as planned (and, if relevant, registered) have been explained.

## Data Availability

There is no data generated for the current study to share.
